# Aqueous extracts of *Liriope platyphylla* induced significant laxative effects on loperamide-induced constipation of SD rats

**DOI:** 10.1186/1472-6882-13-333

**Published:** 2013-11-26

**Authors:** Ji Eun Kim, Young Ju Lee, Moon Hwa Kwak, Jun Ko, Jin Tae Hong, Dae Youn Hwang

**Affiliations:** 1 Department of Biomaterials Science, College of Natural Resources & Life Science, Pusan National University/Life and Industry Convergence Research Institute, Miryang 627-706, Korea; 2 College of Pharmacy and Medical Research Center, Chungbuk National University, Chungju 361-763, Korea; 3 Pusan National University-Wellbeing Products Center, Miryang 627-706, Korea

## Abstract

**Background:**

*Liriope platyphylla* has long been reported as a therapeutic drug for treatment of various human chronic diseases including inflammation, diabetes, neurodegenerative disorders, obesity, and atopic dermatitis. To investigate the laxative effects of *L. platyphylla*, alterations in excretion parameters, histological structure, mucin secretion, and related protein levels were investigated in rats with loperamide (Lop)-induced constipation after treatment with aqueous extract of *L. platyphylla* (AEtLP).

**Methods:**

Alterations on constipation phenotypes were measured in rats with Lop-induced constipation after treatment with AEtLP using excretion parameter analysis, histological analysis, RT-PCR, western blot and transmission electron microscope (TEM) analysis.

**Results:**

The amounts of stool and urine excretion were significantly higher in the Lop + AEtLP-treated group than in the Lop + vehicle-treated group, whereas food intake and water consumption were maintained at constant levels. AEtLP treatment also induced an increase in villus length, crypt layer, and muscle thickness in the constipation model. Total mucin secretion was higher in the Lop + AEtLP-treated group than in the Lop + vehicle-treated group, although mucin secretion per crypt was very similar among all groups. Furthermore, RT-PCR and western blot revealed a dramatic reduction of key factors level on the muscarinic acetylcholine receptors (mAChRs) signaling pathway in the Lop + AEtLP-treated group relative to the Lop + vehicle-treated group. Especially, the accumulation of lipid droplets in enterocytes of crypts following Lop treatment was improved to the level of the No-treated group in response to AEtLP treatment.

**Conclusion:**

These results suggest that AEtLP improves constipation induced by Lop treatment through an increase in crypt layer and stimulation of lipid droplet secretions. These data are the first to show that the laxative effects of AEtLP are closely related to the down-regulation of mAchRs and their downstream signals.

## Background

Constipation is a chronic gastrointestinal disorder characterized by symptoms such as infrequent bowel movements, difficulty during defecation, and sensation of incomplete bowel evacuation [1-3]. Constipation is often caused by insufficient dietary fiber intake, inadequate fluid intake, decreased physical activity, side effects of medication, hypothyroidism, and obstruction by colorectal cancer [4].

Various chemical drugs such as senna, correctol, exlax, senokot, and gaviscon are commonly used to treat constipation, although their use is limited due to high costs and undesirable side effects [5,6]. To date, regulation of the motility of the gastrointestinal tract has been the main focus of constipation treatments. Cisapride was first developed as a promotility agent for treatment of gastric disease, but was later withdrawn because it increased the risk of cardiac arrhythmias [7,8]. Tegaserod, a selective 5-hydroxytryptamine receptor antagonist, is widely used for the treatment of constipation even though it causes coronary artery contraction, coronary spasms, and myocardial infarction [9].

Some plant extracts are also known to exhibit laxative properties based on their ability to increase intestinal motility, frequency and weight of stools and ileum tension. Extract of *Aloe ferox Mill.* has been shown to improve intestinal motility, increase fecal volume, and normalize the body weight of loperamide-induced constipated rats [6]. Moreover, ethanol extracts of agarwood (*Aquilaria sinensis*, *Aquilaria crasna*) leaves are known to increase the frequency and weight of stools, gastro-intestinal transit, and intestinal tension without induction of diarrhea as a side effect [10]. Significant increases in the number, weight, and water content of fecal pellets as well as the intestinal transit length and thickness of the distal colon have also been observed in response to *Ficus carica* paste treatment [11]. Various herbal medicines have recently received attention as novel therapeutic drugs for treatment of constipation.

*L. platyphylla* is a traditional herbal medicine that has long been used for the treatment of asthma as well as bronchial and lung inflammation [12,13]. *L. platyphylla* is also known to potently inhibit airway inflammation and hyperresponsiveness in a murine model of asthma by modulating the relationship between Th1/Th2 cytokine imbalance [13] and atopic dermatitis induced by phthalic anhydride treatment [14,15]. Significant alteration of nerve growth factor (NGF) secretion and its related signaling pathway involving NGF receptors TrkA and p75^NTR^ has been observed in extracts and compounds isolated from *L. platyphylla*-treated PC12 cells [16,17]. *L. platyphylla* has also demonstrated potential for the treatment of obesity and diabetes [18,19]. Gyeongshingangjeehwan, which is primarily composed of *L. platyphylla*, has been reported to prevent obesity and hypertriglyceridemia through the inhibition of feeding and activation of hepatic peroxisome proliferator-activated receptor-alpha in OLETF male rats [19]. Specifically, the homoisoflavone-enriched and LP9M80-H methanol fractions significantly induced insulin secretion and glucose transporter expression through the mitogen-activated protein kinase (MAPK) signaling pathway [18,20]. It is also well known that the roots and extract of *L. platyphylla* contain a wide variety of constituents, although more analyses are needed to verify the correlation between their functions and therapeutic effects. The dry roots of *L. platyphylla* primarily consist of carbohydrates (6.89 g/100 g) and sodium (Na; 6.32 g/100 g), as well as proteins, fat and sugar, while saturated fat, trans-fat and cholesterol are not present. AEtLP extracted from *L. platyphylla* roots contained total saponins (0.56 mg/ml), total soluble solid (31.1 mg/ml), total sugar (15.9 mg/ml), reducing sugar (1.1 mg/ml) and total protein (3.2 mg/ml) [15]. In Korea, the effects of *L. platyphylla* roots on constipation have been recorded in Dong-ui-bo-gam, a medical encyclopedia compiled by the royal physician Heo Jun in 1613, which is on the UNESCO Memory of the World Register [21]. However, scientific evidence of the correlation between *L. platyphylla* roots and constipation have not been presented to date. Therefore, it is worth investigating the laxative effects of AEtLP using an animal model for constipation to verify their mechanism of action under physiological conditions.

Accordingly, the present study was conducted to investigate the laxative effects of AEtLP on Lop-induced constipation in SD rats. The data presented here constitute strong evidence that AEtLP extract is a powerful candidate for alleviation of constipation.

## Methods

### Preparation of AEtLP

The plant materials of *L. platyphylla* were collected from plantations in the Miryang area of Korea at June 2011 and identified by Dr. Cha Shin Woo at the Herbal Crop Research Division, National Institute of Horticultural & Herbal Science. Voucher specimens (WPC-11-010) were deposited at the Functional Materials Bank of the PNU-Wellbeing RIS Center in Pusan National University. Firstly, their roots were dried using a hot-air drier (JSR Instruments, Uttaranchal, India) at 60°C. Six hundred grams of dry roots were reduced to powder using an electric blender and then extracted in 2 L of distilled water, after which the water extract was purified at 100°C for 2 h using circulating extraction equipment (IKA Labortechnik, Staufen, Germany). The calories and main composition of AEtLP had have been measured in previous studies [14,22]. Extract solutions were concentrated into dry pellets using a rotary evaporator (EYELA, Tokyo, Japan) and stored at −80°C until use.

### Care and use of animals

The animal protocol used in this study was reviewed and approved based on ethical procedures and scientific care by the Pusan National University-Institutional Animal Care and Use Committee (PNU-IACUC; Approval Number PNU-2012-0010). Adult SD rats were purchased from SamTacho (Osan, Korea) and handled at the Pusan National University Laboratory Animal Resources Center according to the National Institutes of Health guidelines. All rats were provided with standard irradiated chow diet (Purina Mills, Seoungnam, Korea) *ad libitum* and were maintained in a specific pathogen-free state under a strict light cycle (lights on at 06:00 h and off at 18:00 h) at a temperature of 22 ± 2°C and a relative humidity of 50 ± 10%.

### Induction of constipation and experimental design

Constipation was induced in SD rats by subcutaneous injection of loperamide (4 mg/kg weight) in 0.9% sodium chloride twice a day for 3 days, whereas the non-constipation group was injected with 0.9% sodium chloride alone [6]. For the experiment, 8-week-old SD rats (n = 24) were assigned to either a non-constipation group (n = 12) or a constipation group (n = 12). The non-constipation group was further divided into a No-treated group (n = 6) and an AEtLP-treated group (n = 6). The No-treated group was untreated during the experimental period, whereas the AEtLP-treated group received 15 μl/g body weight of AEtLP (1,000 mg/kg weight) one time. The constipation group was further divided into a Lop + vehicle-treated group (n = 6) and Lop + AEtLP-treated group (n = 6). The Lop + vehicle-treated group received a consistent volume of water via gavage, whereas the Lop + AEtLP cotreatment group received 15 μl/g body weight of AEtLP (1,000 mg/kg weight) one time after the induction of constipation. At 24 h after the AEtLP and vehicle treatment, all animals were sacrificed using CO_2_ gas and tissue samples were acquired and stored in Eppendorf tubes at −70°C until assay.

### Analysis of food intake, water intake, and body weight

Alterations in food intake, water intake, and body weight were measured daily at 10:00 am throughout the experimental period using an electrical balance and a measuring cylinder. All measurements were performed three times to ensure accuracy.

### Measurement of stool parameters and urinary volume

All SD rats were bred in metabolic cages during the experimental period to avoid contamination. The stools and urine excreted from each SD rat were collected at 10:00 am. Stool weight was weighed three times per sample using an electric balance, whereas the water content was determined as the difference between the wet and dry weights of the stool as described previously [6,11]. Changes in the urine volume were measured three times per sample using a cylinder.

### RT-PCR

The frozen transverse colons were chopped with scissors and homogenized in RNAzol B solution (Tet-Test Inc., CS104). The concentration of isolated RNA was then determined by UV-spectroscopy. The expression of the genes was then examined by RT-PCR using 5 μg of the total RNA from each tissue. Briefly, 500 ng of oligo-dT primer [Gibco BRL(18418–012)] was annealed with the template RNA at 70°C for 10 min. The complementary DNA, which served as the template for subsequent amplification, was then synthesized by adding dATP, dCTP, dGTP and dTTP with 200 units of reverse transcriptase and 10 pmole of sense and antisense primers. Next, amplification was conducted by subjecting the samples to 28 cycles of 30 sec at 94°C, 30 sec at 62°C and 45 sec at 72°C in a Perkin-Elmer Thermal Cycler. In each case, negative-RT controls were included to differentiate the DNA and RNA products. RT-PCR was also performed using primers specific to β-actin to ensure the RNA integrity. The primer sequences used to evaluate M2 expression were as follows: sense primer, 5′-CCAGT ATCTC CAAGT CTGGT GCAAG G-3′, antisense primer, 5′-GTTCT TGTAA CACAT GAGGA GGTGC-3′. Also, the primer sequences used to evaluate M3 expression were as follows: sense primer, 5′-GTCAC TTCTG GTTCA CCACC AAGAG C-3′, antisense primer, 5′-GTGTT CACCA GGACC ATGAT GTTGT AGG-3′. The sequences of the β-actin sense and antisense primers were 5′-TGGAA TCCTG TGGCA TCCAT GAAAC-3′ and 5′-TAAAA CGCAG CTCAG TAACA GTCCG-3′, respectively. The level of the PCR products was quantified using a Kodak Electrophoresis Documentation and Analysis System 120 and 1% agarose gels.

### Western blotting

Proteins collected from the transverse colon of No-, AEtLP-, Lop + vehicle- and Lop + AEtLP-treated rats were separated by 4%–20% sodium dodecyl sulfate-polyacrylamide gel electrophoresis (SDS-PAGE) for 3 h, after which the resolved proteins were transferred to nitrocellulose membranes for 2 h at 40 V. Each membrane was then incubated separately with primary antibody, anti-PI-3K (Cell Signaling Technology Inc., Cambridge, MA, USA), anti-PKC (Cell Signaling Technology Inc.) or anti-actin (Sigma-Aldrich, Saint Louis, MO, USA) overnight at 4°C. Next, the membranes were washed with washing buffer (137 mM NaCl, 2.7 mM KCl, 10 mM Na_2_HPO_4_, 2 mM KH_2_PO_4_, and 0.05% Tween 20) and incubated with horseradish peroxidase-conjugated goat anti-rabbit IgG (Zymed Laboratories, South San Francisco, CA, USA) at a dilution of 1:1,000 and room temperature for 2 h. Finally, the membrane blots were developed using Chemiluminescence Reagent Plus kits (Pfizer, New York, NY, USA and Pharmacia, New York, NY, USA).

### Histological analysis

Transverse colons collected from SD rats were fixed with 10% formalin for 12 h, embedded in paraffin wax, and then sectioned into 5 μm thick slices that were stained with hematoxylin & eosin (H&E, Sigma-Aldrich, MO, USA). Morphological features of these sections were observed under light microscopy, after which the villus length, crypt thickness, and muscle thickness were measured using Leica Application Suite (Leica Microsystems, Switzerland).

For mucin staining, transverse colons collected from rats were fixed with 10% formalin for 48 h, embedded in paraffin wax, and then sectioned into 3 μm thick slices that were subsequently deparaffinized with xylene and rehydrated. Next, the samples were rinsed with distilled water and stained with an Alcian blue stain kit (IHC WORLD, MD, USA). Finally, the stained colon sections were observed by light microscopy and the size, number, and morphology of the crypts were measured using Leica Application Suite (Leica Microsystems, Switzerland).

### TEM analysis

The transverse colons collected from rats were fixed in 2.5% glutaraldehyde in 1x PBS buffer, washed, dehydrated with ascending concentrations of ethanol, incubated in 1% OsO_4_ for 1 h at room temperature, and then embedded in Epon812 media (Polysciences, Inc., Germany). Ultra-thin sections (70 nm) were subsequently collected on holy formvar coated grids, contrasted with uranyl acetate and lead citrate, and examined by TEM (Hitachi, Japan).

### Statistical analysis

One-way ANOVA (SPSS for Windows, Release 10.10, Standard Version; SPSS, Chicago, IL, USA) was used to determine whether or not significant differences existed between the AEtLP-treated and vehicle-treated groups or between the non-constipation and constipation groups. All values are reported as the mean ± SD. A *P* value of < 0.05 was considered significant.

## Results

### Body weight and feeding conditions of the constipation rats

**Figure 1:**
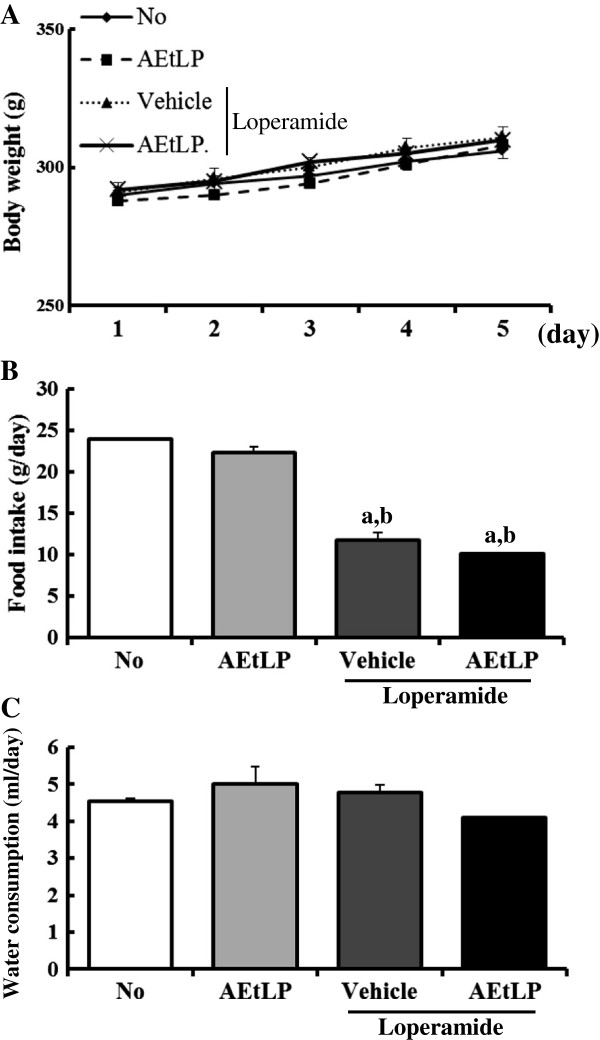
**Effects of AEtLP on body and feeding behavior in Lop-induced constipated rats.** Body weight **(A)**, food intake **(B)**, and water consumption **(C)** were measured at the same time during the experiment. Five to six rats per group were assayed in triplicate for body weight and feeding behavior analysis. Data represent the mean ± SD from three replicates. a, p < 0.05 compared to the No-treated group. b, p < 0.05 compared to the AEtLP-treated group. c, p < 0.05 compared to the Lop + vehicle-treated group.

Body weight did not differ significantly among experimental groups on day 5 (No-treated group; 298 g, AEtLP-treated group; 305 g, Lop + vehicle-treated group; 307 g, Lop + AEtLP-treated group; 302 g), although the Lop + AEtLP-treated group showed a slightly lower body weight than the other groups (Figure 1A). Moreover, SD rats with constipation ate significantly less food than the non-constipation group, while there were no differences between the Lop + vehicle- and Lop + AEtLP-treated groups (Figure 1B). Water consumption also did not change in the constipation group or non-constipation group. Furthermore, no significant increase in water consumption was detected in the Lop + AEtLP-treated group (Figure 1C). Taken together, these results show that AEtLP treatment did not induce any alteration of body weight, food intake or water consumption.

### Stool and urine excretion

**Figure 2:**
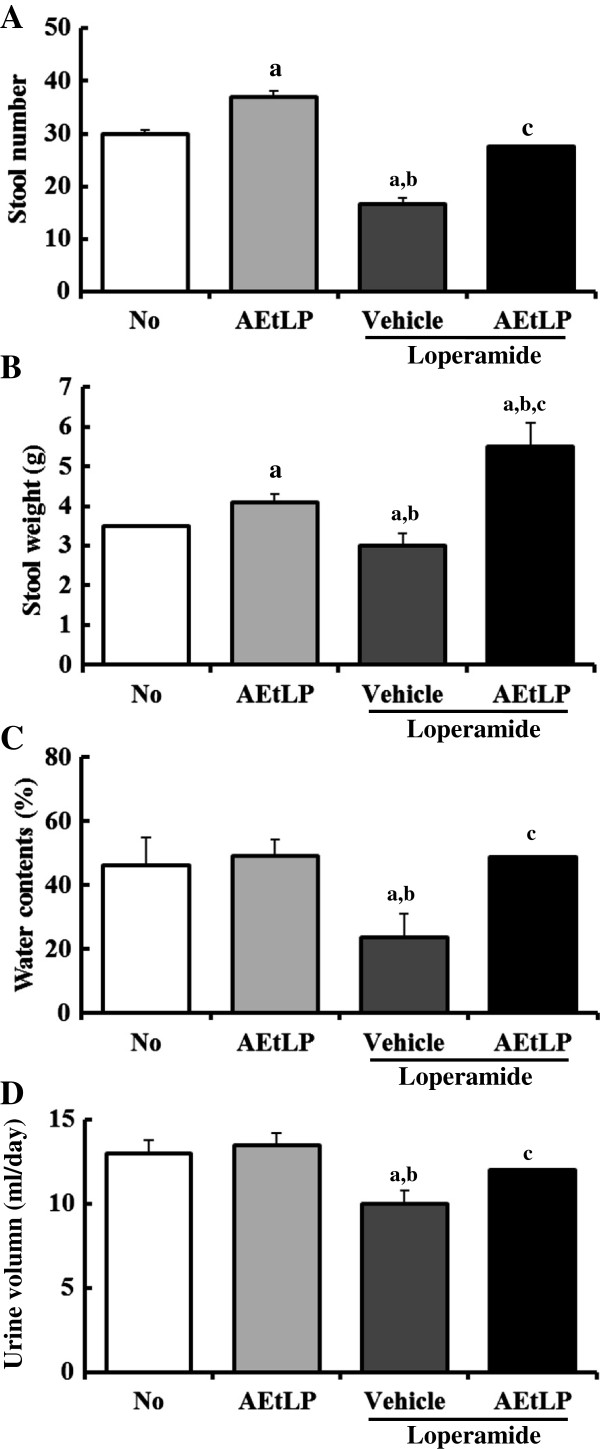
**Effects of AEtLP on stool and urine secretion in Lop-induced constipated rats.** Stool number **(A)**, stool weight **(B)**, and urine volume **(D)** were measured immediately using an electric balance and measuring cylinder. Water content of stools **(C)** was determined by drying stools for 12 h. Five to six rats per group were assayed in triplicate for excretion analysis. Data represent the mean ± SD from three replicates. a, p < 0.05 compared to the No-treated group. b, p < 0.05 compared to the AEtLP-treated group. c, p < 0.05 compared to the Lop + vehicle-treated group.

The effects of any treatment on constipation are generally determined based on altered excretion from laboratory animals. To investigate the laxative effects of AEtLP on stool and urine excretion, alterations in excretion parameters were measured in No-, AEtLP-, Lop + vehicle- and Lop + AEtLP-treated SD rats. Excretion volumes of stool and urine were significantly reduced after administration of Lop, while they increased slightly in the AEtLP-treated group. However, these levels in the Lop + AEtLP-treated group were almost recovered to those in the No-treated group (Figure 2A, B and D). Indeed, the weight of stool in the Lop + AEtLP-treated group was approximately twice that of the No-treated group (Figure 2B). Furthermore, the water content of stool in the Lop + vehicle-treated group was roughly 45-55% lower than that in the No-treated group. However, after Lop and AEtLP cotreatment this level was elevated significantly to that of the No-treated group (Figure 2C). These results suggest that AEtLP treatment could improve Lop-induced constipation in SD rats through enhancement of stool and urine excretion.

### Histological alteration of transverse colon

**Figure 3:**
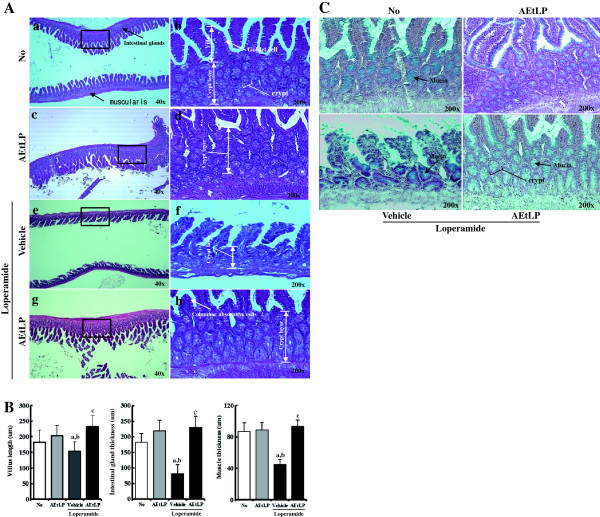
**Effects of AEtLP on histological parameters and mucin secretion in Lop-induced constipated rats. (A)** H&E-stained sections of transverse colons rats from the No-treated group (a and b), AEtLP-treated group (c and d), Lop + vehicle-treated group (e and f), or Lop + AEtLP-treated group (g and h) were observed at two different magnifications using a light microscope. **(B)** Villus length, crypt layer thickness, and muscle thickness are presented as graphs. **(C)** Mucin secretion from transverse colon. Blue indicates mucin stained with alcian blue at pH 2.5. Five to six rats per group were assayed in triplicate by H&E and alcian blue staining. Data represent the mean ± SD from three replicates. a, p < 0.05 compared to the No-treated group. b, p < 0.05 compared to the AEtLP-treated group. c, p < 0.05 compared to the Lop + vehicle-treated group.

To investigate whether AEtLP treatment could induce alteration of the histological structure of the transverse colon, the villus length, crypt layer thickness, and muscle thickness were measured in transverse colons of rats in the three groups following H&E staining. The average length of the villus was significantly shorter in the Lop + vehicle-treated group than the No-treated group. Following Lop and AEtLP cotreatment, this level greatly increased by more than 60% when compared with the Lop + vehicle-treated group (Figure 3A and B). Furthermore, alteration of crypt layer thickness and muscle thickness was very similar to that of villus length. In the Lop + vehicle-treated group, the crypt layer thickness and muscle thickness were dramatically reduced when compared with those in the No-treated group. However, in the Lop + AEtLP-treated group, thickness levels were recovered to those in the No-treated group, although the thickness of the crypt layer increased by 30-35% relative to that of the No-treated group (Figure 3A and B). These results show that AEtLP induced increases in villus length, crypt layer thickness, and muscle thickness in the transverse colon of constipated SD rats.

### Effect of AEtLP treatment on mucin secretion

Lop-induced constipation has been shown to induce a decrease in mucus production from crypt epithelial cells [23]. To evaluate the effects of AEtLP treatment on mucin production, we measured the level of mucin production in Lop-induced constipation rats after AEtP treatment. The total level of mucin was reduced significantly in the Lop + vehicle-treated group relative to that in the No-treated group, while these levels were dramatically recovered to up to 70-80% of those in the No-treated group after Lop and AEtLP cotreatment. However, these changes were due to alteration of crypt layer thickness rather than alteration of secretion level per crypt (Figure 3C). Overall, these findings indicate that AEtLP treatment may induce an increase in total mucin secretion from villus region, although the level of mucin secretion per crypt remained nearly unchanged.

### Effect of AEtLP treatment on expression of mAchRs and their downstream proteins

**Figure 4:**
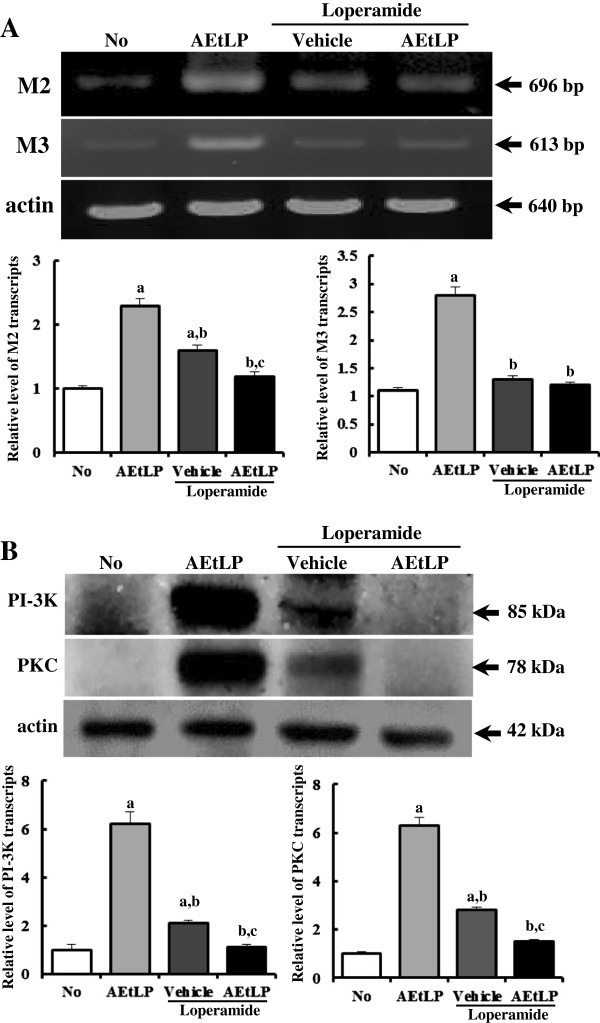
**Level of mAChR and their downstream signal.** Alteration of the level of mAChR M2 and M3 transcripts was measured by RT-PCR **(A)**, while PI-3 K and PKC level was determined using Western blot **(B)**. The level of mAChR M2 and M3 transcripts in transverse colons was measured by RT-PCR using specific primers. After the intensity of each band was determined using an imaging densitometer, the relative levels of PI-3 K and PKC protein were calculated based on the intensity of actin protein. Five to six rats per group were assayed in triplicate by RT-PCR and Western blotting assays. Data represent the mean ± SD of three replicates. a, p < 0.05 compared to the No-treated group. b, p < 0.05 compared to the AEtLP-treated group. c, p < 0.05 compared to the Lop + vehicle-treated group.

Little is known about the proteins associated with constipation, although mAchR M2 and M3 has been reported to play a role in the contraction of smooth muscle [24]. To investigate whether AEtLP treatment could affect the regulation of proteins related to muscle contraction, alteration of mAchR M2, mAchR M3, PI-3K and PKC expression was observed in the transverse colons of constipation rats using specific primers and antibody. The expression patterns of these four factors were very similar among all groups. RT-PCR analysis revealed that the levels of mAchR M2 and M3 transcripts in the AEtLP-treated group increased by 212% and 280% relative to those in the No-treated group. Following Lop treatment, these levels greatly recovered to those in No-treated rats, although their level was not completely restored (Figure 4A). Furthermore, the band intensities for PI-3 K and PKC, a member of the downstream pathway of mAchR M2 and M3, were in good agreement with the RT-PCR results. The Lop + vehicle-treated group displaying symptoms of constipation showed higher expression of PI-3K and PKC proteins in the transverse colon than the No-treated group. However, the expression levels of PI-3K and PKC in the Lop + AEtLP-treated group were recovered to those in the No-treated group (Figure 4B). Taken together, these results suggest that the laxative effects induced by AEtLP treatment are correlated with the down-regulation of mAchRs and their downstream signal in the transverse colons of constipated SD rats induced by Lop treatment.

### Effect of AEtLP treatment on ultrastructure of the transverse colon

**Figure 5:**
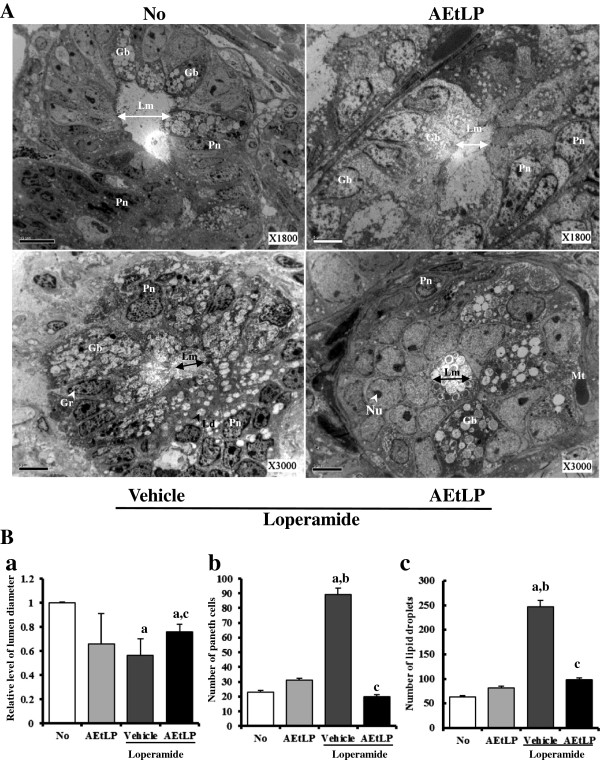
**TEM image of transverse colon. A**. The ultrastructure of the crypt in the No-, AEtLP-, Lop + vehicle-, and Lop + AEtLP-treated group was viewed by TEM at x1800 or x3000 magnification. **B**. Diameter of crypt lumen **(a)**, the number of paneth cells **(b)** and lipid droplets **(c)** were measured using Leica Application Suite (Leica Microsystems, Switzerland). Five to six rats per group were assayed in triplicate by TEM analysis. Data represent the mean ± SD from three replicates. a, p < 0.05 compared to the No-treated group. b, p < 0.05 compared to the AEtLP-treated group. c, p < 0.05 compared to the Lop + vehicle-treated group. Lm, lumen of crypt; Gb, goblet cells; Pn, paneth cells; Gr, granule cells; Ld, lipid droplets; Nu, nucleus; Mt, mitochondria.

To investigate the effects of AEtLP treatment on the ultrastructure of the transverse colon, ultrastructural changes in cells and organelles were detected by TEM analysis. In the No-treated group, the Crypt of Lieberkuhn showed a ring structure in which enterocytes, goblet cells, and paneth cells surrounded a lumen at the center. This structure in the AEtLP-treated group was similar to that of the non-treated group, although the number of lipid droplets was slightly greater in the AEtLP-treated group. Following Lop treatment, the ultrastructure of the crypt changed dramatically in every aspect. The diameter of the crypt lumen was also smaller in the Lop + vehicle-treated group than in the No-treated group, although Lop + AEtLP treatment did not induce any significant alteration of lumen diameter (Figure 5A and Ba). Furthermore, changes in the number of paneth cells were very similar to those in the lumen diameter (Figure 5A and Bb). However, Lop + AEtLP treatment had a significant effect on the number of lipid droplets in the lumen. The crypt lumen maintained an empty state in the No-treated group and a small number of lipid droplets in the Lop + vehicle-treated group. Conversely, high accumulation of lipid droplets was detected in the crypt lumen of the Lop + AEtLP-treated group. This was especially true in the Lop + vehicle-treated group, where the abundance of lipid droplets and granules increased greatly in enterocytes or paneth cells, while the levels in goblet cells remained the same. In the Lop + AEtLP-treated group, the lipid droplets and granules clearly disappeared from enterocytes and paneth cells (Figure 5A and Bc). The cells in the Lop + AEtLP-treated group were also more round and broad than those in the No-treated group (Figure 5A). These results show that AEtLP effectively restored alteration of the ultrastructure, including increasing the abundance of lipid droplets and granules in the crypt of the transverse colons of constipated rats.

## Discussion

Herbal plants and medicinal foods have recently received increased attention as novel therapeutic drugs for the treatment of constipation and its related diseases [10,11,25]. In an effort to develop and investigate drugs for the treatment of constipation, we investigated the therapeutic effects of AEtLP on Lop-induced constipated SD rats. The results clearly demonstrated that AEtLP has laxative effects, including elevation of stool and urine volumes and the recovery of histological changes induced by Lop in the transverse colon. Our data are the first to demonstrate that the laxative effects of AEtLP are tightly correlated with the down-regulation of mAchRs and their downstream signal, alteration of the ultrastructure, and mucin secretion in the transverse colon.

Several chemical compounds including Lop and morphine are widely applied to induce constipation in laboratory animals. Among these, Lop is well known to stimulate the extension of stool evacuation time and the delay of intestinal luminal transit through inhibition of water secretion [26] and smooth movement in the intestinal wall [27,28]. Furthermore, Lop has been used to induce constipation in a variety of studies to determine the cause of constipation and identify novel compounds with therapeutic effects [6,11,25]. Although the dose and time for Lop treatment have varied among studies, constipation was successfully induced by treatment with 1.5 – 3 mg/kg body weight of Lop for 3–7 days [6,11,25,29,30]. In the present study, we used Lop to induce constipation and observed the human-like symptoms of constipation in SD rats injected with 4 mg/kg of Lop without any specific problems. Furthermore, Lop and racecadotril have been successfully applied to decrease the risk of dehydration and systemic or metabolic diseases such as diarrhea. Loperamide is a mu-opiate-receptor agonist that extends the orocecal and colonic transit times by increasing gut activity, disarranging electrical activity in the gut and delaying the passage of fluid through the small intestine, although it has some side effects such as severe allergic reaction, toxic megacolon, constipation, decreased urination and stomach bloating [31,32]. Racecadotril is an indirect delta receptor agonist that accompanies antisecretory activity without an increase of activity in gut muscle [33,34].

It should be noted that there are some limits and restrictions to the clinical translation of results obtained from Lop induced rats to human conditions, even though the experimental procedure for rat models which is easy and reproducible without negative side effects or histologically detectable damages are widely used for the evaluation and development of therapeutic drug [35]. Chronic constipation is categorized into three groups by assessment of colonic transit and anorectal function; normal transit or irritable bowel syndrome, pelvic floor dysfunction (functional defecatory disorders) and slow transit constipation [36]. Among patients with chronic constipation, the most prevalent form is normal transit (59%), followed by functional defecatory disorders (25%), slow transit (13%) and a combination of defecatory disorders and slow transit (3%) [37]. However, models of pharmacological constipation induced by Lop can only simulate slow transit constipation showing less daily fecal excretion, lower water content, lower numbers of fecal pellets and thinner fecal mucus [23,29,38]. Therefore, our results obtained from the Lop-induced constipation model cannot completely translate all forms of chronic constipation detected in human patients. Additionally, further studies are needed to develop a novel model of phenotypes of all classes of chronic constipation using genetic engineering technology and transient injection of chemical substances.

Food intake and water consumption are considered to be important factors for evaluation of constipation symptoms and therapeutic effects. However, Lop-induced constipation models can have several different effects on food intake and water consumption. In some cases, administration of Lop induces decreases in food intake and water consumption [6], whereas some studies have shown that Lop treatment does not induce any change in food intake or water consumption [11]. In our study, food intake was reduced in response to Lop administration, while water consumption was maintained. These findings are in agreement with those of a study conducted by Shimotoyodome et al. [23], although the rate of decrease in food intake differed among several studies. Furthermore, a variety of responses regarding food intake and water consumption in constipation models are induced by several herbal medicines and foods. Aqueous leaf extract of *Aloe ferox* Mill. only induces enhancement of water consumption, whereas food intake is consistently maintained without any significant alteration [6]. In addition, food intake and water consumption can be significantly decreased by the administration of *Ficus carica* paste for 4 weeks [11]. In the present study, AEtLP treatment did not induce any significant alteration of food intake or water consumption, although a slight decrease in water consumption was observed. These differences might be due to factors such as the innate tastes of the herbal medicines and foods used in each study.

A significant reduction in fecal excretion in Lop-induced rats is considered one of the key markers of constipation in most constipation studies. Previously, stool-related factors such as pellet number, weight, and water content were shown to be dramatically decreased in rats upon administration of Lop [6,10,11,29]. However, these alterations were significantly recovered by plant extracts with laxative effects. *Aloe ferox* Mill., a widely used medicinal plant with healing properties, has been shown to improve the number, water content, and weight of stools in Lop-induced rats in a dose-dependent manner [6]. Furthermore, leaf extracts of *Mareya micrantha* increased stool output of rats relative to a control group at doses of 200 and 400 mg/kg [25]. In the present study, a similar effect on stool-related factors was observed in Lop + AEtLP-treated rats (Figure 2).

Furthermore, histological studies have demonstrated significant alterations in the intestines of constipation-induced animals. The thickness of the distal colon layer decreases significantly upon Lop treatment [11], and the average thickness of the mucus layer is thinner in Lop-treated rats than in control rats [23]. As shown in Figure 3, similar results were observed in our study, such as decreases in crypt layer and muscle thickness in Lop-treated SD rats, although the detection site differed between them [11,23]. Furthermore, our data indicated that villus length decreased significantly in the transverse colons of Lop-induced constipated rats. These histological changes have been shown to be recovered by herbal medicines and foods having laxative effects. AEtLP treatment showed similar results in the present study as those observed in response to treatment with *Ficus carica* paste, which induced increases in the thickness of the distal colon and areas of crypt epithelial cells in a dose-dependent manner. Specifically, the thickness of the crypt layer was restored to that of the No-treated group [11].

Mucin is an important component of luminal mucus, which protects the colorectal mucosa from a variety of mechanical and chemical damage [39,40]. Secretion of mucin is usually detected by staining with alcian blue at pH 2.5. In this study, crypt epithelial cells and lumens were strongly stained with alcian blue in No-treated rats (Figure 3), which is agreement with the results of previous reports [23,41] and may validate the present histochemical results. For example, Lop administration induces reduction of mucin storage in crypt epithelial cells by decreasing both the thickness and amount of the luminal mucus layer [23]. Similar effects on mucin secretion were detected in the present study. As shown in Figure 3, the total level of mucin was significantly reduced in Lop-treated SD rats, but was recovered in Lop + AEtLP-treated rats. However, the amount of mucin per crypt lumen was maintained in all groups. Taken together, these findings indicate that one of the main reasons for the decrease in total mucin was the reduced thickness of the crypt layer.

mAChRs are a type of ACh receptor that mediate cholinergic signaling and are expressed on the surfaces of certain neurons and other cells, including heart and smooth muscle cells [41,42]. These cells act as main receptors and are activated by acetylcholine secreted from postganglionic fibers in the parasympathetic nervous system. Upon activation, signals are transferred to G protein-receptor complexes via cytoplasmic domain interactions, stimulating phospholipase c, which then cleaves PIP2 into IP2 and DAG. The enhanced DAG then induces the activation of PKC, which is involved in receptor desensitization, modulation of membrane structure events, regulation of transcription, immune responses, regulation of cell growth, and learning and memory [43,44]. mAChRs are classified into five subtypes (M1-M5) according to their tissue distribution and signal transduction mechanism [45]. The role of these receptors has received great attention as they have emerged as key therapeutic drugs [46]. Among the five mAChR subtypes, M1, M2, and M3 significantly influence intestinal activity, including motility and secretion [47]. mAChR M2 has the following indirect function in cholinergic contraction: (1) mAChR M2 stimulation may reverse the relaxation of smooth muscle induced by isoproterenol via inhibition of adenylate cyclase [24], (2) mAChR M2 stimulation may participate in regulation of intracellular signaling originating from mAChR M3 [24], and (3) mAChR M2 may play a role in papillary dilation rather than papillary contraction as suggested by studies of mAChR M2^−/−^M3^−/−^ mice [48]. In this study, we selected mAChR M2 and M3 as a target protein to investigate the molecular mechanism behind the laxative effects of AEtLP. We successfully detected the level of mAChR M2 and M3 transcripts in the transverse colon by RT-PCR as shown in previous studies, although the origin of the samples and detection method used in the analyses was not the same [47]. Furthermore, significant alterations of the level of mAChR M2 transcripts were observed under abnormal conditions, including ischemia, neuropathic pain, arrhythmia, and diabetes [49-52]. The results presented herein are the first to show that the level of mAChR M2 and M3 transcripts dramatically increased in the transverse colon during AEtLP treatment or Lop-induced constipation. These increases in mAChR M2 and M3 transcripts were decreased by AEtLP treatment. Furthermore, the expression pattern of PI-3K and PKC in the down-stream signaling pathway was very similar to that of mAChR M2 and M3. The increase in PI-3 K and PKC expression induced by Lop administration was inhibited by AEtLP treatment. Taken together, the alteration of mAChR M2 and M3 signaling pathway observed in the present study provide information that can be used in future investigations of the causes of constipation and selection of targets for constipation treatment.

The Crypt of Lieberkühn is an intestinal gland located in the epithelial lining of the small intestine and colon. The crypt is composed of three major cell types, enterocytes, goblet cells, and paneth cells. Enterocytes are simple columnar epithelial cells responsible for the digestion and absorption of ions, sugars, peptides, amino acids, lipids, vitamins, electrolytes, and water. Paneth cells are able to secrete various enzymes, including sucrase and maltase, along with enteropeptidase. Goblet cells are glandular, simple, columnar epithelial cells that secrete mucin dissolved in water to form mucus with apocrine and merocrine patterns [53,54]. As shown in Figure 5, our results provide the first evidence that the ultrastructural changes in the three crypt cells are tightly correlated with the progression and recovery of Lop-induced constipation. After induction of constipation, lipid droplets containing mucin accumulated in the cytoplasm of goblet cells and enterocytes, whereas abundant granules were present in paneth cells. However, Lop + AEtLP treatment stimulated the secretion of lipid droplets into the crypt lumen in both goblet cells and enterocytes.

## Conclusion

The results of this study suggest that AEtLP could induce the recovery of stool number, water content, and urine quantity while enhancing villus length, crypt layer, and muscle thickness. In addition, these results provide evidence that the laxative effects of AEtLP may be mediated by the regulation of mAchRs and their downstream signal as well as the secretion of lipid \droplets. Furthermore, these findings indicate that AEtLP could be considered one of the therapeutic candidates for the treatment of constipation, although many additional studies are required to confirm this.

## Abbreviations

Lop: Loperamide; AEtLP: Aqueous extract of *L. platyphylla*; TEM: Transmission electron microscope; mAChRs: Muscarinic acetylcholine receptors; PI-3K: Phosphoinositide 3-kinase; PKC: Protein kinase C; RT-PCR: Reverse transcription-polymerase chain reaction.

## Competing interests

The authors have no competing interests to declare.

## Authors’ contributions

JEK, YJL, MHK, JK and DYH participated in the design of the study, sample preparation, animal experiments and data analyses. JTH helped with data analysis and manuscript preparation. All authors read and approved the final manuscript.

## Pre-publication history

The pre-publication history for this paper can be accessed here:

http://www.biomedcentral.com/1472-6882/13/333/prepub
